# Enhanced Homing of Mesenchymal Stem Cells Overexpressing Fibroblast Growth Factor 21 to Injury Site in a Mouse Model of Traumatic Brain Injury

**DOI:** 10.3390/ijms20112624

**Published:** 2019-05-28

**Authors:** Rami Ahmad Shahror, Ahmed Atef Ahmed Ali, Chung-Che Wu, Yung-Hsiao Chiang, Kai-Yun Chen

**Affiliations:** 1 Ph.D. Program for Neural Regenerative Medicine, College of Medical Science and Technology, Taipei Medical University and National Health Research Institutes, Taipei 110, Taiwan; rami.shahror@yahoo.com (R.A.S.); ychiang@tmu.edu.tw (Y.-H.C.); 2 Center for Neurotrauma and Neuroregeneration, Taipei Medical University, Taipei 110, Taiwan; johnwu@tmu.edu.tw; 3 TMU Neuroscience Research Center, Taipei Medical University, Taipei 110, Taiwan; ahmed.atef@tmu.edu.tw; 4 Translational Laboratory, Department of Medical Research, Taipei Medical University Hospital, Taipei 110, Taiwan; 5 Department of Neurosurgery, Taipei Medical University Hospital, Taipei 110, Taiwan; 6 Department of Surgery, School of Medicine, College of Medicine, Taipei Medical University, Taipei 110, Taiwan

**Keywords:** regenerative medicine, mesenchymal stems cells, fibroblast growth factor 21, traumatic brain injury, cell tracking, cell homing, cell therapy, gene therapy

## Abstract

Mesenchymal stem cells (MSCs) are emerging as a potential therapeutic intervention for brain injury due to their neuroprotective effects and safe profile. However, the homing ability of MSCs to injury sites still needs to be improved. Fibroblast Growth Factor 21 (FGF21) was recently reported to enhance cells migration in different cells type. In this study, we investigated whether MSCs that overexpressing FGF21 (MSC-FGF21) could exhibit enhanced homing efficacy in brain injury. We used novel Molday IONEverGreen™ (MIEG) as cell labeling probe that enables a non-invasive, high-sensitive and real-time MRI tracking. Using a mouse model of traumatic brain injury (TBI), MIEG labeled MSCs were transplanted into the contralateral lateral ventricle followed by real-time MRI tracking. FGF21 retained MSC abilities of proliferation and morphology. MSC-FGF21 showed significantly greater migration in transwell assay compared to control MSC. MIEG labeling showed no effects on MSCs’ viability, proliferation and differentiation. Magnetic resonance imaging (MRI) revealed that FGF21 significantly enhances the homing of MSC toward injury site. Histological analysis further confirmed the MRI findings. Taken together, these results show that FGF21 overexpression and MIEG labeling of MSC enhances their homing abilities and enables non-invasive real time tracking of the transplanted cells, provides a promising approach for MSC based therapy and tracking in TBI.

## 1. Introduction

Traumatic brain injury (TBI) is the most common form of head injury and is estimated to result in death or hospital admission for more than 10 million people worldwide annually [1,2]. TBI can be divided into mild, moderate, and severe TBI. Cognitive and memory impairments are frequently associated with TBI [3]. Deficits in learning and memory have been reported in several cognitive domains, including executive function, attention, working memory, episodic memory, verbal learning, and processing speed [4,5]. In both animal models and human studies, cognitive impairment following TBI has been correlated with neural atrophy and progressive cell death in the hippocampus and frontal cortex.

Mesenchymal stem cells (MSCs) transplantation produced improvements in TBI experimental studies [6,7,8]. MSCs have the capacity to secrete trophic factors that activate endogenous neurorestorative processes and/or mediate neuroprotection due to their anti-inflammatory effects within the injured brain [6,7,8,9]. The ease with which MSCs can be isolated, and expanded in vitro show their feasibility as potential approach to repair or reconstitute brain injury. A clinical trial has demonstrated that transplantation of bone marrow mononuclear cells in adults with severe TBI insult is a safe and feasible option for cell-based therapy in humans [10].

The ability of MSCs to migrate and identify the injury site determines their therapeutic efficacy. The physiological and regulatory mechanisms underlying MSC homing is complex and not yet fully understood [11]. Despite their well-documented ability to migrate to sites of injury after TBI, the majority of infused MSCs fail to survive due to the harsh microenvironment, lack of oxygen and inadequate nutrients which can minimize the therapeutic benefit of MSCs [12,13,14]. An in vitro study showed that priming MSCs with lithium improved migration and homing abilities by inducing MMP-9 (a matrix metallopeptidase) and CXCR-4 (a chemokine receptor), respectively [15]. Preconditioning MSCs with lithium and valproate enhanced the survival of MSCs by inducing robust changes in the expression of genes involved in trophic effects in the brain after intranasal delivery in N171-82Q Huntington’s disease mice [16]. One of the most prominent trophic proteins induced by lithium and valproate co-treatment in neurons and MSCs is fibroblast growth factor-21 (FGF21) [16,17].

Although FGF21 is well known for its actions as peripheral metabolic regulator, particularly in response to fasting, novel roles of its action in central nervous system (CNS) injuries were investigated. An in vitro study showed that FGF21 can activate cell-survival related protein kinase Akt-1 and inhibit glycogen synthase kinase 3 (GSK3) causing robust neuroprotective properties against glutamate excitotoxicity in primary brain neurons [17]. Systemic administration of recombinant human FGF21 (rhFGF21) reduced brain edema and protect against blood-brain barrier disruptions 24 h after TBI in experimental model [18]. Furthermore, in vivo study showed that peripherally-derived FGF21 improved remyelination in a mouse model of toxin-induced demyelination [19]. While FGF21 effects on cells migration in vitro has been reported in fibroblasts and cardiomyocytes, no study has reported the effect of FGF21 on MSC migration in vitro or on homing to brain injury site in vivo [20,21,22].

Given these promising protective and homing-enhancing features of FGF21, we used a genetically engineered MSCs that overexpress FGF21 and then assessed their homing abilities after contralateral intracerebroventricular (ICV) transplantation of MSCs overexpressing FGF21 (MSC-FGF21) in a mouse model of TBI. However, the lack of non-invasive methods for stem cell tracking after transplantation in a longitudinal fashion without postmortem histology represents a serious challenge for stem cell therapy in clinical settings.

Advances in nanotechnology have produced non-invasive approaches for stem cells tracking in vivo. Magnetic nanoparticles can be efficiently internalized by stem cells and generate strong magnetic resonance imaging (MRI) contrast [23]. An appropriate MRI contrast agent is superparamagnetic iron oxide (SPIO) nanoparticles, which have been successfully used for labeling different mammalian cell types [24,25,26]. Stem cells labeling with SPIO and MRI can be used to quantify stem cells that are able to migrate from the site of transplantation toward the injury site, and to visualize their biodistribution in a longitudinal fashion in vivo. SPIO are compose of a negatively charged nanosized core of iron oxide coated with a hydrophilic shell. The coating of SPIO is generally formed to overcome it tendency to aggregate [27]. MSC labeled with SPIO coated with biocompatible dextran had no significant effects on cell viability, proliferation, or functionality [28]. SPIO can be internalized into stem cells and cluster in endosomal and/or lysosomal compartments and cause magnetic field distribution in MRI scanning [29,30].

In this work, we thoroughly studied the effect of the SPIONs in MSCs that overexpress FGF21 using commercially available dextran-coated SPIO, Molday ION™ EverGreen (MIEG; BioPAL, Worcester, MA, USA), as a direct cell-labeling agent for tracking stem cells in vivo by MRI. MIEG was evaluated to determine the labeling efficiency. The effects of MIEG in MIEG-labeled stem cells viability, proliferation and stemness were evaluated. Finally, MSC-FGF21 labeled with MIEG was real-time tracked after transplantation in a mouse TBI model using a 7-Tesla (7T) MRI to evaluate their homing feature to the injury site.

## 2. Results

### 2.1. FGF21 Enhances MSC Migration In Vitro

**Figure 1:**
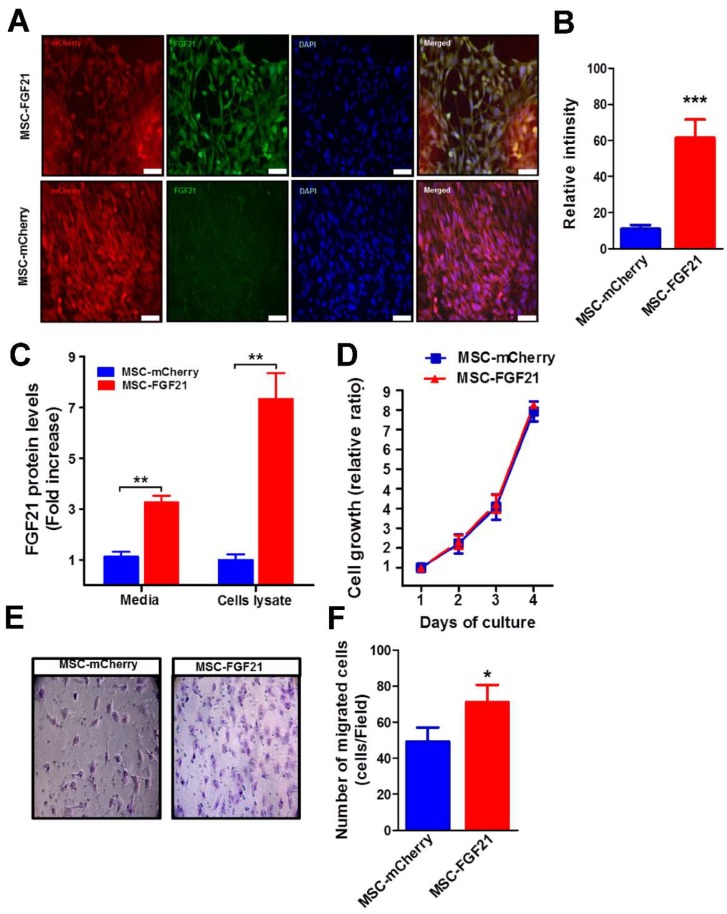
Characterization and in vitro migratory effects of Mesenchymal stem cells (MSCs) that overexpressing Fibroblast Growth Factor 21 (FGF21) and mCherry protein (MSC-FGF21). (**A**) Representative images from immunofluorescence microscopy was used analyze the expression of FGF21 and transfection efficiency using anti-FGF21 primary and anti-rabbit secondary antibodies conjugates Alexa 488 (green). DNA was counterstained with 4′,6-diamidino-2-phenylindole (DAPI, blue). (**B**) Quantification of FGF21 expression levels based on Immunofluorescence intensity of FGF21 using ImagJ and ElISA (**C**). (**D**) Cell growth was assessed daily for MSCs that overexpress only mCherry protein (MSC-mCherry) and MSC-FGF21 for four days using a trypan blue dye exclusion assay. (**E**) Representative images (200× magnification) of migrated MSC-mCherry and MSC-FGF21 in transwell assay. (**F**) The migrated cells are highlighted by crystal violet staining (purple) and were quantified; the MSC-FGF21 showed a significant higher migration ability compared to MSC-mCherry. Data are mean ± S.E.M. of triplicate values from three separate experiments. * *p* < 0.05, ** *p* < 0.01, *** *p* < 0.005. (**A**,**B**) Scale bar represents 50 mm.

Both MSC-FGF21 and the control MSC-mCherry highly expressed the reporter protein (mCherry), confirming the stable expression of mCherry reported gene (Figure 1A). No change was observed in the cell morphology or growth capacity for both MSCs due to mCherry and/or FGF21 overexpression (Figure 1A). 

FGF21 overexpression was confirmed by FGF21 immunofluorescence relative intensity that quantified using ImageJ (Figure 1A,B) and ELISA (Figure 1C). MSC-FGF21 exhibited a seven-fold (p < 0.001) increase in FGF21 level compared to the MSC-mCherry (Figure 1C). FGF21 overexpression has no effect on MSCs proliferation (Figure 1D). FGF21 has been reported to play a role in cell migration and invasion [20,21,22,31]. We therefore examined if FGF21 overexpression in MSCs could also increase the MSCs migration in vitro. We found that MSC-FGF21 displayed a significant increase (p < 0.05) in migration compared to MSC-mCherry using the Transwell assay (Figure 1E,F).

### 2.2. MIEG Nanoparticle Size and Distribution

**Figure 2:**
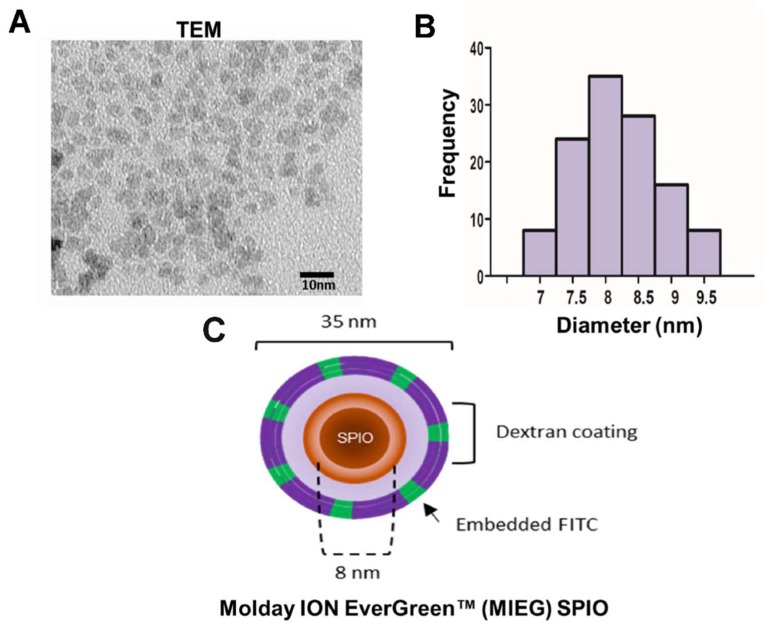
Characterization of MoldayION™ EverGreen (MIEG). (**A**) Transmission electron microscopy (TEM) of MIEG at concentration of 200 μg/mL, (**B**) size distribution of MIEG, and (**C**) a schematic illustration of MIEG. Scale bar 10 nm.

SPIO uptake by cells may depend on the SPION coating, the surrounding medium, and SPION aggregation behavior. The stability of MIEG that would be used for labeling in the environment (DMEM) at a high concentration (200 μg/mL) was investigated by TEM using a Hitachi HT-7700 electron microscope (Hitachi High-Tech, Tokyo, Japan) operating at an 80–200 kV accelerating voltage, which measures variation in core size and was used to evaluate the aggregation behavior. The TEM results show that the morphology of the MIEG is nearly spherical with some agglomerates (Figure 2A). The mean nanoparticles size was approximately 8 nm (Figure 2B), which is smaller than the hydrodynamic diameter (33.6 ± 5.1 nm) suggested by the manufacturer. However, the visual TEM examination revealed the only core, whereas the hydrodynamic diameter is the sum of the core size and the molecules layer.

### 2.3. Efficient In Vitro Uptake of MIEG by MSC

**Figure 3:**
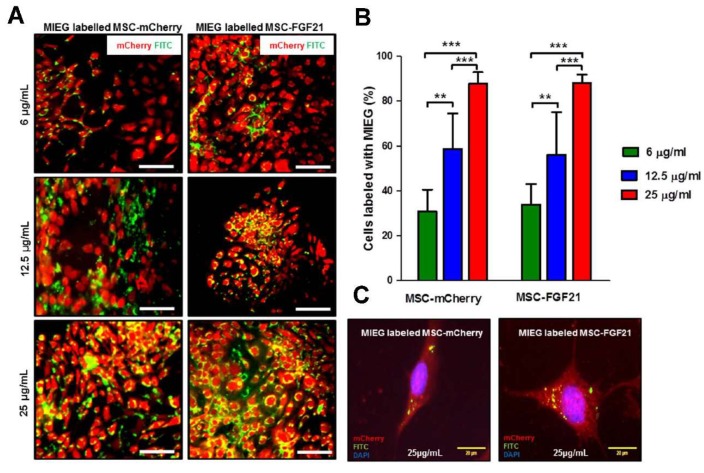
In vitro labeling of MSCs with MIEG. (**A**) Presence of MIEG in MSCs was detected by fluorescence microscope. Representative images fluorescence microscope of MSC-mCherry and MSC-FGF21 incubated with different concentrations of MIEG for 24 h. (**B**) Labeling percentage in monolayer cultures of MSC-FGF21 and MSC-mCherry exposed to 6, 12.5, and 25 μg/mL of MIEG for 24 h. (**C**) Representative fluorescence microscope image demonstrating the characteristic intracellular distribution of MIEG (green) in MSCs after 24 h. Data represent mean ± S.E.M. of triplicate values from three separate experiments. ** *p* < 0.01, *** *p* < 0.005. Scale bar 50 μm unless stated otherwise.

The presence of iron nanoparticles within the cells was confirmed using a fluorescence microscope and staining with Prussian blue under a light microscope (Figure 3A and Figure S1). Efficient MIEG uptake was not observed when MSC-mCherry or MSC-FGF21 was incubated with 6 μg/mL MIEG for 24 h (Figure 2A,B). However, both cells were efficiently up taking the MIEG when incubated with 12.5 and 25 μg/mL MIEG labeling medium (Figure 3A,B). As shown in Figure 3B, the uptake efficacy was significantly higher when MSC-mCherry or MSC-FGF21 was incubated with 25 μg/mL compared to 12.5 (*p* < 00.005) or 6 (*p* < 00.005) μg/mL MIEG for both cells. To further understand where the particles are located within the cells, the high magnification image from fluorescence microscopy of MSC-FGF21 and MSC-mCherry labeled with MIEG are shown in Figure 3C. The iron particles were compartmentalized within endosomes in the cell cytoplasm. The small green spheres within the vesicles are the iron oxide core of the MIEG nanoparticles.

### 2.4. Assessment of Biological Effects of MIEG-Labeled MSCs In Vitro

Although MIEG is classified as a non-hazardous substance by the manufacturer, no data were available regarding their toxicological effects. Since MIEG is a novel and relatively new product, new toxic features may be found that induce cell damage. To the best of our knowledge, no studies have been published for cytotoxicity measurements of MIEG in MSC in vitro.

**Figure 4:**
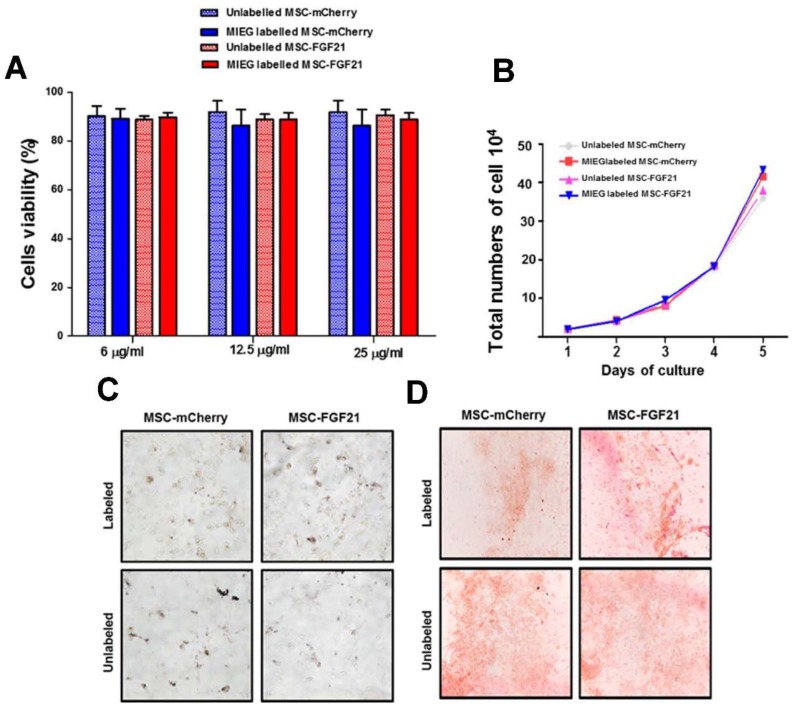
Evaluation of MSC viability and proliferation after MIEG labeling. Viability of MSCs incubated with MIEG 24 h.(**A**) No change in MSCs viability was observed for MSCs labeled with 6, 12.5, or 25 μg/mL MIEG for 24 h. (**B**) Proliferation curve of unlabeled controls and labeled MSCs in triplicate at a MIEG concentration of 25 μg/mL. (**C**) Representative images (200× magnification) of Oil Red O staining of unlabeled controls and labeled MSCs at a MIEG concentration of 25 μg/mL for adipogenic differentiation to determine lipid droplet content. (**D**) Representative images (200× magnification) of Alizarin red S staining of unlabeled controls and labeled MSCs at a MIEG concentration of 25 μg/mL for osteogenic differentiation to detect bone mineralization. Quantified data are mean ± SEM of triplicate values from three separate experiments.

To investigate the optimal labeling concentration, we assessed cellular viability in Trypan blue exclusion. Neither MSC-FGF21 nor MSC-mCherry display any changes in viability with increasing MIEG concentration up to 25 μg/mL (Figure 4A). The proliferation abilities of both cell types were not affected by 24 h incubation with 25 μg/mL MIEG (Figure 4B). Therefore, a MIEG concentration under 25 μg/mL is safe for labeling MSC-FGF21 and MSC-mCherry with a 24 h incubation time, so is the preferable choice for labeling both cell lines.

To investigate whether MIEG labeling affected the multipotency of MSC to differentiate into traditional mesenchymal lineages, we evaluated the adipogenic and osteogenic differentiation potency of MIEG labeled MSC-mCherry and MSC-FGF21 in vitro using differentiation media with lineage-specific induction supplement. Both MSC-mCherry and MSC-FGF21 labeled with 25µg/mL of MIEG showed efficient adipogenic differentiation potency as unlabeled cells that revealed from red colored lipid vacuoles following Oil red O staining (Figure 4C). Similarly, MIEG labeling did not affect the osteogenic differentiation potency of MSC-mCherry and MSC-FGF21 (Figure 4D).

### 2.5. In Vitro MRI of the MSC Phantoms

**Figure 5:**
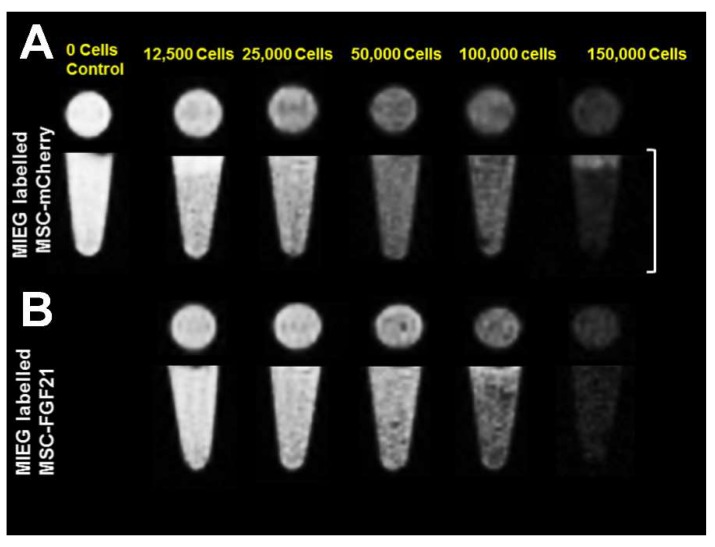
Magnetic resonance imaging (MRI) of a phantom injected with 100 μL of gelatin containing MIEG-labeled MSCs. The images were captured using T2*weighted imaging (T2*WI) based on spin echo. (**A**) MIEG-labeled MSC-mCherry and (**B**) MSC-FGF21 are visualized as hypointense areas in the isointense milieu. Images captured are presented in axial projection (upper panels) and coronal projection (lower panels). The scale bars represent 10 mm.

Representative MRI images of a phantom of 100 μL gelatin contain different amounts of MIEG-labeled MSC-mCherry or MIEG-labeled MSC-FGF21 obtained using T2* weighted imaging (T2*WI) based on spin echo are shown in Figure 5. Given the ability SPIO to disturb the magnetic field, resulting in a T2* relaxation time reduction that appears as hypointense microspheres in MRI, MIEG-labeled MSC-mCherry and MSC-FGF21 produce a MRI signal reduction visualized as dark hypointense microspheres. MRI images of MSC-mCherry and MSC-FGF21 MIEG-labeled with 25 μg/mL MIEG revealed that approximately 12,500 cells per 100 mL were still detectable.

### 2.6. FGF21 Overexpression Promotes Rapid, Targeted, and Stable Homing of MIEG-Labled MSCs In Vivo in TBI Mouse Brain

**Figure 6:**
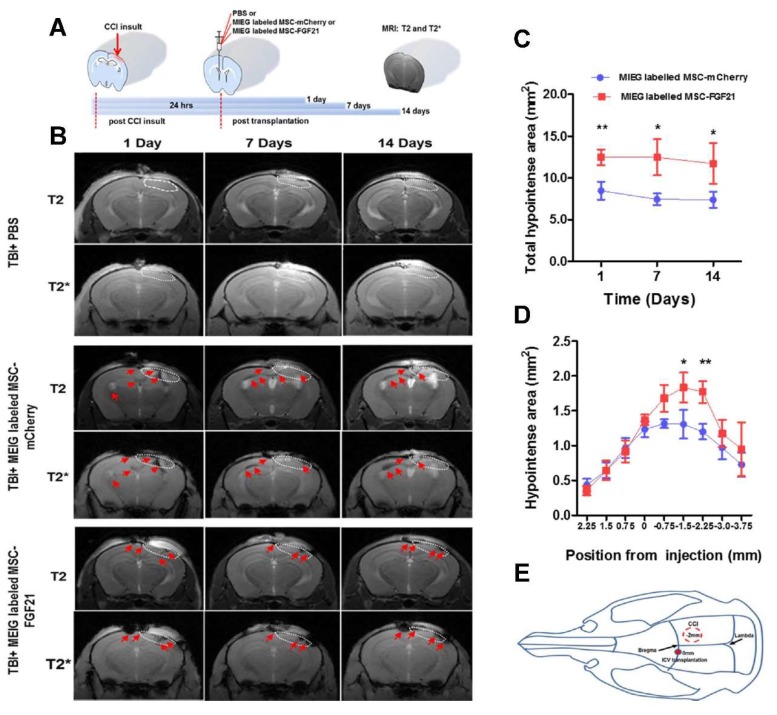
Detecting and tracking the homing of MIEG-labeled MSC in vivo. (**A**) Graphic and schematic illustration timeline of real-time T2* and T2WI MRI scanning of mice brains following controlled cortical impact (CCI) injury and MIEG-labeled MSCs transplantation. (**B**) Representative images of T2* and T2WI MRI scanning at different time point showing the homing of transplanted MIEG-labeled MSC-FGF21 and MSC-mCherry as hypointense signals (red arrows) at the injury site (dotted lined area). Time course evaluation of total hypointense areas generated by MIEG-labeled MSC-FGF21 and MSC-mCherry. (**C**) Time course evolution of total hypointense area showed no significant differences between 1, 7 and 14 days, but showed differences between the two groups. (**D**) Distributions of hypointense areas generated by MIEG-labeled MSC-FGF21 and MSC-mCherry 14 days post-transplantation in mice brains expressed as distances anterior (positive) and posterior (negative) to the transplantation site. (**E**) Graphic illustration of CCI injury site and MSC transplantation coordinate used in the current study. Quantified data are mean ± SEM (*n* = 7–8 animals per group). * *p* < 0.05, ** *p* < 0.01 compared between MIEG labeled MSC-FGF21 and MIEG labeled MSC-mCherry treated TBI animals.

To determine whether MIEG-labeled MSCs could migrate to injury sites and be tracked in a longitudinal fashion after ICV transplantation contralateral to brain injury, we used mice subjected to controlled cortical impact (CCI) insult. After 24 h from CCI insult, a dose of 1.5 × 10^5^ MSC-mCherry or MSC-FGF21 MIEG-labeled with 25 μg/mL were transplanted in the contralateral lateral ventricle and MRI visualization was conducted at different time points post-transplantation (Figure 6A). The control group was subjected to CCI insult and injected with PBS alone.

MRI scanning revealed dark hypointense areas in T2 and T2* images that corresponded to the MIEG-labeled MSC at the injury sites in the MIEG-labeled MSC-mCherry-treated group as well MIEG-labeled MSC-FGF21-treated group for the entire study duration (Figure 6B). No recognizable hypointense signal was detected at site of injury in the PBS-treated group for the 14 days post-transplantation (Figure 6B). T2* images showed enhanced contrast compared to T2. In both MIEG-labeled-MSCs-treated groups, MSC homing to injury sites was observed as hypointense signals as early as one day post-transplantation. This was expected since cell migration to injuries has been reported to occur as early as 24 h post stem cell transplantation [32]

In MIEG-labeled MSC-FGF21-treated mice, cell homing to the injury site remained stable and constant with no significant reduction in total hypointense area for the entire 14 days post-transplantation. In MIEG-labeled MSC-mCherry-treated mice, however, less homing was observed and MSC was still detectable in the contralateral hemisphere (Figure 6B). The total hypointense areas were calculated in T2*WI and were significantly higher in MIEG-MSC-FGF21-treated animals compared to MIEG-labeled MSC-mCherry-treated animals (1 day, *p* < 0.01; 7 days, *p* < 0.05; and 14 days, *p* < 0.05) (Figure 6C).

MSC homing was further analyzed by calculating the position of the hypointense areas in the brains of MSC-treated animals based on T2*WI. In both groups, the hypointense area caused by MIEG-labeled MSCs peaked at 0.75–2.25 mm posteriorly to the transplantation site (position 0.0 mm) (Figure 6D), which coincides with of the core of CCI insult site (position –2.0 mm) (Figure 6E). However, MIEG-labeled MSC-FGF21 showed significant and enhanced homing to the core of the injury site at positions –0.75 mm (*p* < 0.05) and –2.25 mm (*p* < 0.01) compared to MIEG-labeled MSC-mCherry.

**Figure 7:**
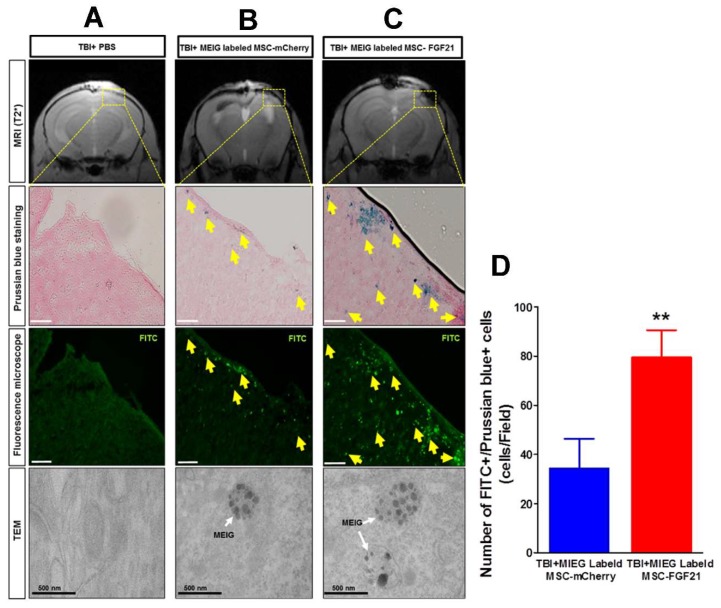
Ex vivo histological analysis. (**A**–**C**) Prussian blue staining combined with Fluorescein isothiocyanate (FITC) reactivity 14 days post-transplantation. (**C**) In MSC-FGF21-treated animals, the iron nanoparticles (blue) in Prussian Blue staining and FITC reactivity that represent MIEG are overlapped and located at the position of MRI hypointensities at the injury site. Similarly, in MSC-mCherry-treated animals, the iron nanoparticles are located to the same areas as FITC and MRI hypointensities and were less pronounced compared to MSC-FGF21 (**B**). (**D**) Quantative analysis of the Prussian blue and FITC positive cells numbers at the injury site (**A**) In PBS-treated animals, iron staining, FITC reactivity, and MRI hypointensities are almost absent at injury site. TEM imaging was used to further investigate the intracellular iron presence at the injury site for all groups (lower panel). The dotted boxed represents the injury site; yellow arrows show Prussian Blue and FITC-positive cells; white arrows show the intracellular MIEG at the injury site. Scale bar is 50 mm unless stated otherwise. Quantified data are mean ± SEM (*n* = 4 animals per group) of. ** *p* < 0.01 compared between MIEG labeled MSC-FGF21 and MIEG labeled MSC-mCherry treated TBI animals.

Histological analyses of the perfused brains were performed 14 days post-MSC transplantation to confirm the homing of MSCs. Prussian Blue staining revealed iron-positive cells that coincided with the T2* hypointense areas that were observed at the injury site in brains of MIEG-labeled MSC-FGF21- and MSC-mCherry-treated animals (Figure 7B,C). Since MIEG has FITC embedded in the shell of the nanoparticles, fluorescence microscopy revealed that the Prussian Blue-positive cells overlapped FITC-positive cells at the injury site of both treated groups, further confirming the homing of labeled MSC-FGF21and MSC-mCherry. As was expected from the T2* images, the injury site in brains of MIEG-labeled MSC-FGF21-treated animals showed a significant increase (*p* < 0.01) of the Prussian Blue and FITC positive cells numbers than MSC-mCherry-treated animals (Figure 7D). These results further confirmed the enhanced homing MSC-FGF21 at the injury sites. Further analysis of the injury site by TEM imaging confirmed the presence of MIEG nanoparticles at the injury site of both MSCs-treated groups. In PBS-treated animals, at injury site, Prussian Blue or FITC-positive cells were absent (Figure 7A).

### 2.7. The Survival and Proliferation Ability of Transplanted MSCs at the Injury Site

**Figure 8:**
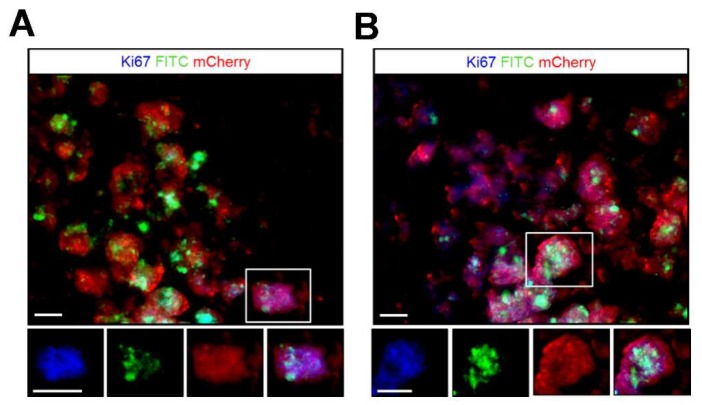
The survival and proliferation of the transplanted MIEG labeled MSCs at the injury site. 14 days post transplantation, the survival of the MIEG labeled MSC-mCherry (**A**) and MIEG labeled MSC-FGF21 (**B**) at the injury site was examined via mCherry immunoreactivity (red) that overexpress by the viable MSCs. The proliferation ability of the viable MSCs was examined via Ki67 immunoreactivity (blue). The majority of FITC (green) positive cells are viable MSCs. Examples of Ki67+/mCerry+/FITC+ cells are indicated by white box and represented by single channel images (lower panel). Scale bars: 100 μm.

The survival and proliferation ability of the transplanted MSCs at the injury site was examined through immunohistochemical staining using antibodies targeting the cell cycle antigen Ki67 and mCherry that overexpressed by the viable MSC-FGF21 and MSC-mCherry at 14 days post-transplantation (Figure 8). MSC-FGF21 and MSC-mCherry were survived at the injury site and able to proliferate, for 14 days at least. Furthermore, FITC signals that generated by MIEG were colocalized with the mCherry positive cells suggesting that the FITC and Prussian positive cells and concede hypointense areas at the injury site (Figure 7) are viable MSCs.

## 3. Discussion

Clinical interest has been focused on MSC therapy as a potential therapeutic approach for TBI, where the treatments available are limited. MSCs transplantation has been shown to mitigate brain injuries by intracranial or intravenous delivery [33,34]. However, the therapeutic efficiency and success of such approaches are highly dependent on the proliferation and homing of the transplanted MSCs to the target tissue [35]. The major findings of our study are as follows: (1) the overexpression of FGF21 significantly enhances migration of MSCs in vitro with no effects on their proliferation, (2) MIEG labeling of MSCs exerts neither cytotoxic, proliferative anti-stemness effects on MSC-FGF21 or MSC-mCherry, (3) MIEG labeling of MSC-FGF21 and MSC-mCherry enables non-invasive in vivo real-time MRI tracking of the cells in TBI mice model, and (4) MIEG-labeled MSC-FGF21 exhibit rapid and enhanced homing toward the injury site in TBI mice brain.

The potential role of FGF21 in TBI therapy has been reported in animal models [18,19]. The neuroprotective properties of FGF21 against glutamate excitotoxicity in primary neurons has been reported via Akt-1 activation and the GSK3 inhibition axis [17]. Although the role of FGF21 in cell migration in vitro has been reported in various cells, no study has reported the effect of FGF21 on MSC migration in vitro or on homing to brain injury site in vivo [20,32]. FGF21 exhibited a very short half-life of only one to two hours and can be readily cleared from the brain by circulation [36]. In this study, we used genetically engineered MSC to continuously overexpress murine FGF21 by lentiviral vectors, thus maintaining high and steady levels of FGF21 in TBI brains for the entire study period. Our results demonstrate that the MSCs that overexpress FGF21 exhibited a seven-fold (*p* < 0.001) higher expression of FGF21 than control MSC-mCherry (Figure 1C). The majority of both cell types were positive for fluorescence reporter protein mCherry with no change in their growth rate. Subsequently, we investigated the ability of FGF21 to promote cell migration in vitro. Our results demonstrated that overexpression of FGF21 significantly (*p* < 0.05) enhanced migration MSC in vitro in the Transwell assay (Figure 1E,F).

For in vivo studies of MSC homing to the injury site, it is essential to establish a non-invasive and in vivo imaging protocol, such as MRI, and real-time tracking of transplanted stem cells at the injury site. To detect the transplanted stem cells via MRI, SPIO have been widely used to label the cells prior to transplantation due to their MRI contrast enhancement properties and safety. SPIO have been used in both preclinical and clinical applications [37,38]. However, a study demonstrated that high intracellular iron oxide nanoparticle concentrations can causes proliferation impairment as a result of cytoskeletal changes in the human dental pulp stem cells in vitro [39]. Here, we demonstrated that MSC-FGF21 and MSC-mCherry were efficiently labeled with MIEG at a concentration of 25 μg/mL after 24 h incubation with no significant alteration in their viability, proliferation, morphology. Both MSC-mCherry and MSC-FGF21were differentiated into osteoblasts regardless of whether or not they were MIEG labeled. The osteogenic differentiation was demonstrated by Alizarin Red S staining (Figure 4F). Similar results obtained from adipogenic differentiation demonstrated by Oil red O staining (Figure 4E). These results are in agreement with previous published report [30]. Collectively, these results provide in vitro evidence that MIEG labeling at concentration of 25 μg/mL has no adverse effects on cell growth, viability or the stemness of MSC-mCherry or MSC-FGF21.

We used MRI to validate whether MIEG labeled MSCs were detectable in vitro. We demonstrated that low cells number as 12,500 cells (<10% of the number that used in the in vivo study) of MIEG-labeled MSC-mCherry and MSC-FGF21 were able to be detected by in vitro T2* MRI (Figure 5). We used in vivo MRI to track in longitudinal fashion the transplanted MIEG labeled MSCs and evaluate their homing efficiency in TBI mice model. The experimental scheme is outlined in Figure 6A. Our results demonstrated by T2 andT2*WI MRI (Figure 6B) that MIEG-labeled MSC-FGF21 resulted in dark hypointense areas in the site of injury shown at entire study period (Figure 6C). Similar results were detected in mice treated MIEG-labeled MSC-mCherry (Figure 6B,C). However, less hypointese area were detected at injury site at the same period (Figure 6D). PBS treated-control mice did not develop hypointense signals at the injury site. The histological analysis confirmed the spatial migration and homing of MIEG-labeled MSC-FGF21 and further confirm that the hypointense areas at injury site are generated by the MIEG labeling (Figure 7). Previous studies reported that SPIOs can be released by the dead labeled cells post transplantation and internalized by surrounding activated microglia that might results in false positive MRI signals in vivo [40,41]. In this regard, our results demonstrate that the majority of FITC positive cells at the injury site were viable MSCs in both treated group (Figure 8) and able to proliferate, suggesting that the detected MRI signal are correspond to the viable MSCs. These results in agreement with a previous study that examined the long term fate of SPIO labeled neural stem cells after transplantation [24].

Although the specific mechanisms whereby FGF21 promotes MSCs homing abilities in the current study has been undefined, a previous study found that FGF21 mimics treatment promotes human umbilical vein endothelial cells (HUVECs) through the activation of eNOS/PI3K/AKT pathways. Furthermore, FGF21 found to promote cells migration through the β-catenin signaling cascade in feedback regulatory loop and regulate the activity of c-Jun N-terminal kinase (JNK) signaling, a key regulator in fibroblast cell migration, in skin fibroblasts [21,22]. However, these studies did not investigate the effects of FGF21 overexpression on MSCs migration and homing in vivo. Further investigations are warranted to completely clarify the underlying mechanisms whereby FGF21 overexpression in MSCs enhances the homing abilities.

Collectively, these results demonstrating the enhanced and targeted homing abilities of MSC-FGF21 and feasibility of MIEG labeling for non-invasive, real-time in vivo MRI tracking of the transplanted MSCs. The limitation of the present study is lack of behavioral and neurological tests for functional outcome assessment in the TBI-mice model. Thus, future studies should further evaluate the therapeutic effects of MSC-FGF21 transplantation on TBI-induced functional impairments.

For the first time, we found that FGF21 overexpression in MSC promotes targeted cells homing to the injury site of TBI in an animal model. The MIEG labeling of MSC-FGF21 and MSC-mCherry enables real-time non-invasive in vivo MRI tracking of transplanted cells. We have laid the foundation for a novel approach to improve the therapeutic effects of MSC transplantation by enhanced the homing and enable non-invasive, real-time tracking of MSC for TBI treatment in the future.

## 4. Materials and Methods

### 4.1. Cell Culture

We used murine bone marrow derived MSC that purchased from Invitrogen (Carlsbad, CA, USA) and stably overexpress FGF21 (MSC-FGF21) and control MSC-mCherry, prepared and generously provided by De-Maw Chuang (Intramural Research Program, National Institute of Mental Health, National Institutes of Health, Bethesda, MD, USA). The cells were cultured in DMEM/F-12 medium with GlutaMAX™-I (Gibco, Invitrogen, Carlsbad, CA, USA), supplemented with 10% fetal bovine serum (FBS; Gibco, Invitrogen), and 5 μg/mL gentamycin. Cells were cultured at 37 °C and 5% carbon dioxide. The MSC had been stained positive for the cell surface protein markers CD29, CD34, CD44, and Sca-1 (>70%), and negative for CD117 (<5%) in flow cytometry assays performed by the manufacturer (Invitrogen). MSC-FGF21 and MSC-mCherry were sorted using fluorescence-activated cell sorting (FACS) to isolate a highly enriched population of MSCs expressing the mCherry reporter gene. The mCherry-positive cells were then cultured, expanded, and used for subsequent studies. MSCs from passages 3–7 and 3–5 were used for in vitro and in vivo experiments respectively.

### 4.2. Immunofluorescence

To confirm the overexpression of FGF21 in MSC, we used immunofluorescence. The MSCs were grown directly onto glass coverslips until reaching 80% confluence. The coverslips were washed in 1× phosphate buffered saline (PBS), and the cells were fixed in paraformaldehyde (2% in 1× PBS) at 4 °C for 15 min. Endogenous peroxidase activity was quenched by the incubation of the slides in 4% hydrogen peroxide solution in 1× PBS for 40 min, followed by one hour blocking with 3% normal goat serum and 3% Bovine serum albumin (BSA). The slides were then incubated overnight at 4 °C with diluted primary antibodies in blocking buffer that included rabbit anti-FGF21(1:200; Aviscera Bioscience, Santa Clara, CA, USA). After a series of PBS washing, slides were then incubated with appropriate Alexa Fluor 488 conjugated-secondary antibodies (1:1000; Invitrogen, Carlsbad, CA, USA) for 1 h at room temperature. The slides were then washed with cold PBS (3 times), and then coverslipped with Fluoroshield mounting medium containing 4′,6-diamidino-2-phenylindole (DAPI) (GeneTex, Taipei, Taiwan).

### 4.3. Enzyme-Linked Immunosorbent Assay (ELISA) for FGF-21

FGF-21 protein levels in the lysates of MSCs-FGF21 and MSC-mCherry and their media after 24 h incubation were determined using a mouse/rat FGF-21 ELISA Kit (Aviscera Bioscience, Santa Clara, CA, USA) according to the manufacturer’s protocol. Briefly, 96-well microplates pre-coated with IgG against mouse FGF-21 were incubated with samples or FGF-21 standards for 2 h at room temperature with shaking. After washing, biotinylated antibodies against mouse FGF-21 was added to each well and then the plate was covered, sealed and incubated for 2 h on a microplate shaker at room temperature. After repeated washings to remove unbound antibody, streptavidin conjugated to horseradish peroxidase was added to each well. The plate was further incubated for 60 min followed by washings. Tetramethylbenzidine (TMB) substrate solution was added to each well followed by 8 min incubation. Stop solution was added to each well to allow color change from blue to yellow. The optical density (OD) was detected at 450 nm using a microplate reader. A standard curve was created by blotting the OD of the standard dilutions versus its corresponding FGF21 concentrations, and sample FGF21 levels were then calculated.

### 4.4. Transwell Migration Assay

MSC-mCherry or MSC-FGF21 cells (4 × 10^4^ cells/cm^2^) were inoculated into the upper layer of a transwell insert (Corning, Corning, NY, USA) in a serum-free medium with 10% FBS containing the medium at the bottom layer. After incubating for 24 h at 37 °C, cells at the upper layer of the membrane were scraped, and migrated MSCs at the lower layer were stained with crystal violet and photographed under a microscope. The number of stained MSCs was counted manually in 5 fields under a light microscope at 200× magnification.

### 4.5. MIEG Nanoparticles Characteristics and Stability in Culture Medium

We used a commercial SPIO, MIEG (CL-50Q02-6A-51, BioPAL, Worcester, MA, USA). MIEG is a dextran-coated SPIO that has a hydrodynamic diameter of 35 nm, positive charge (zeta potential +31 mV), with R1 and R2 of 30.4 and 75.8 mM^–1^·s^–1^, respectively, and an iron concentration of 2 mg/mL. MIEG is a homogeneous fluorescent nanoparticle, tagged with fluorescein isothiocyanate (FITC), and designed to label cells efficiently and simply without addition of a transfection agent. A study of the stability of MIEG was performed in DMEM/F-12 (Gibco, Invitrogen) with an iron concentration of 100 µg/mL. Core size measurements were recorded using a transmission electron microscope (TEM).

### 4.6. MIEG Labeling of MSCs

Prior to labeling, MIEG was diluted at various concentrations (6, 12.5, and 25 µg/mL) in DMEM F-12 medium that did not contain FBS and was gently shaken for 10 min at room temperature. The solutions containing solution were added to MSC cultures and maintained in incubator at 37 °C and 5% carbon dioxide for 24 h.

### 4.7. Prussian Blue Staining

Prussian blue staining was used to detect iron within the MSCs in culture after MIEG labeling, and to track labeled MSCs ex vivo post-transplantation in TBI mice brain. This method relies on the reduction of ferric iron to the ferrous state to generate a blue ferrocyanide precipitate. For in vitro staining, MSCs were cultured in 12 wells and labeled with MIEG, then washed twice with PBS and fixed for 15 min in 4% paraformaldehyde at 37 °C. After fixation, the cells were washed twice with PBS and incubated with Perls’ reagent (20% potassium ferrocyanide and 20% hydrochloric acid) for 15 min at room temperature. Cultures were then washed twice in distilled water and counterstained with nuclear fast red. MSC were observed using light microscopy.

For ex vivo detection of MIEG-labeled MSCs, brains sections (12 μm) were washed twice with PBS and incubated with Prussian blue staining with shaking. After 30 min, sections were rinsed in water and counterstained with nuclear fast red, dehydrated, and covered.

### 4.8. Labeling Efficiency

To quantitatively evaluate the labeling efficiency, MSCs were assessed by counting cells that were positive for Prussian Blue staining and the presence of FITC, which indicated MIEG presence within the cell. Briefly, 10 fields of view were randomly chosen for counting Prussian blue positive cells under a light microscope, and the labeling efficiency was calculated using the following equation: Labeling Efficiency = (Prussian blue positive cell number/whole cell number) × 100%.

### 4.9. MIEG-Labeled MSC Viability/Cytotoxicity

The effect of MIEG on viability of MSCs was determined using the Trypan Blue exclusion assay. MSCs were cultured in 6-well plate in duplicate and incubated with MIEG media or un-supplemented media (unlabeled). Media were aspirated without disrupting the cellular monolayer cells and washed with PBS and trypsinized. The harvested cells were centrifuged at 3000 rpm for 5 min then suspended in 1× PBS at the concentration of 5 × 10^5^/mL and mixed with 0.4% trypan blue dye at a 1:1 ratio. Duplicates of 10 µL of this mixture were loaded into a hemocytometer, after which cells were counted. Cells with an intact membrane excluded the dye and were considered live cells. The percentage of live and dead cells was determined.

### 4.10. Kinetics of Proliferation Labeling with MIEG

Approximately 1 × 10^5^ MSC labeled with MIEG were incubated in triplicate at iron concentrations of 6, 12.5, and 25 µg/mL for 24 h, followed by two PBS washes and incubated in fresh DMEM/F-12 medium supplemented with 10% FBS and maintained in incubator at 37 °C and 5% carbon dioxide for 7 days. Next, cells were washed with PBS and harvested with TrypleLe (Gibco, Invitrogen). The harvested cells were centrifuged at 3000 rpm for 5 min then suspended in 1× PBS then mixed with trypan blue dye at 1:1 ratio. Duplicates of 10 µL of the cell mixture for each replicate were loaded into the hemocytometer. The total cells from each point in the curve are expressed by mean of triplicates.

### 4.11. Osteogenic and Adipogenic Differentiation for Osteogenic Differentiation

To investigate whether MIEG labeling can affect MSCs multipotency to differentiate traditional mesenchymal lineages, differentiation was conducted. StemPro^®^ Adipogenesis and StemPro^®^ Osteogenesis Differentiation Kits (Gibco, Invitrogen) were used and with some modification to manufacture recommendation. Briefly, MSC-mCherry and MSC-FGF21 were seeded at density of 0.5 × 10^4^ cells/cm^2^ in a 12-well plate with DMEM-F12 with 10% FBS and 5 μg/mL gentamicin1. Following 24h incubation with 25 µg/mL of MIEG, the cells washed twice with PBS and incubated with MSC Basal Medium for two days. Then cells incubated with MSC Basal Medium supplemented with StemPro^®^ Osteogenesis Supplement or StemPro^®^ Adipogenic differentiation Supplement. The medium was changed twice a week for 3 weeks, and cells were subjected to Alizarin Red S (purchased from Sigma-Aldrich, St. Louis, MO, USA) staining for osteogenic differentiation or Oil Red O (purchased from Sigma-Aldrich) staining for adipogenic differentiation using commercial staining solutions.

### 4.12. Surgery and Transplantation Procedures

Surgeries were performed on 6–8-week-old wild-type C57B/L male mice that were obtained from the National Laboratory Animal Center in Taipei, Taiwan. The animals were housed in cages under a standard 12-h light/dark cycle with food and water available ad libitum, and their body weights were measured bi-weekly. All procedures were approved by the Institutional Animal Care and Use Committee and with the approval of the Ethics Committee for animal use in Taipei Medical University (approval no. LAC-2018-0189; 16.07.2018).

TBI was induced using a CCI device to induce a unilateral, moderate injury. Prior to CCI induction, mice were anesthetized by an intraperitoneal (i.*p*.) injection of zolazepam (50 mg/kg) and rompun (20 mg/kg) and positioned in a stereotaxic frame (Stoelting, Wood Dale, IL, USA) prior to TBI. A 4 mm craniotomy was performed under sterile conditions at coordinate of 2.0 mm posterior to the bregma and 2.0 mm lateral to the sagittal suture. After carefully exposing the intact dura, a mild to moderate TBI was induced using an impacting tip with a diameter of 3 mm driven by pneumatic piston at a velocity of 5 m/s, a depth of 1 mm, and a dwell time of 250 ms. Then, the scalp was sutured, and the mouse was monitored throughout the recovery phase and body temperature of 36–37 °C was maintained using heating a pad. Then, 24 h after TBI induction, the animals were injected with 1.5 × 10^4^ cells/5 μL of MSC-mCherry or MSC-FGF21 labeled with MIEG at concentration of 25 μg/mL for 24 h on the contralateral side of the injury hemisphere at coordinates of 0 mm caudal to Bregma and 1 mm lateral to the midline. Using an automatic syringe pump system (Singa, Taoyuan, Taiwan) and a Hamilton syringe (27 gauges), 5 μL of cell suspension was ICV-infused at a depth of 3 mm beneath the dura mater. The cell suspension was infused over 5 min at a rate of 1 μL/min, and the needle was left in place for another five minutes. The TBI control animals were infused with vehicle (in this case, PBS). The scalp was then sutured. Animals were kept at 37 °C during the recovery phase and closely monitored.

### 4.13. In Vivo and In Vitro MRI

MRI was employed to locate the MIEG labeled-MSC-FGF21 and MSC-mCherry post-transplantation via the effect of the SPIO particles on imaging contrast. We divided 24 mice into 3 equal groups for MRI scanning: (1) TBI-injured animals + PBS, (2) TBI-injured animals + MIEG labeled-MSC-mCherry, and (3) TBI-injured animals + MIEG-labeled-MSC-FGF21.

All animal imaging sessions were performed using a 7T scanner (PharmaScan 70/16; Bruker Biospin, Ettlingen, Germany). The animals were maintained under anesthesia using 1.5–2% isoflurane and kept under regular room air during image acquisition. Body temperature was monitored and maintained during the scanning. Coronal images were obtained with a T2WI based on spin echo sequence (TR 2500 ms; TE 33 ms; 8 average), and a T2*WI based on spin echo sequence (TR 1500 ms; TE 2.8 and 4 average). The slice thickness of 0.75 mm was constant across the optimized sequences. The mice were first scanned 24 h after injection with T2WI images to evaluate the anatomy and T2*WI images to visualize MIEG-positive cells. The animals were then scanned every week until sacrifice with T2WI and T2*WI MRI. All image matrices were zero-filled to 128 × 128 for further analyses. We used the segmentation command in ITK-SNAP software to quantify the area and position of the dark hypointense areas [42].

For in vitro MRI, samples of different cells number of MSC-mCherry or MSC-FGF21 labeled with MIEG at concentration of 25 μg/mL for 24 h embedded in 100 μL of 2% gelatin and underwent MRI using the T2*WI spin echo sequence protocol. Both coronal and axial images were captured.

### 4.14. Histological Analysis

Animals anesthetized with a mixture of zolazepam/rompun underwent transcardial perfusion with PBS followed by chilled 4% paraformaldehyde in PBS. The brains were then carefully removed from the skull and post-fixed overnight at 4 °C. Following post-fixation, brains were dehydrated in 20% and then 30% sucrose for cryoprotection. Thereafter, the tissues were incubated in optimal cutting temperature (OCT) resin (Sakura Finetek USA Inc., Torrance, CA, USA) and 12-μm frozen sections were collected on microscope slides. Adjacent slices were stained with Perl’s Prussian blue stain to determine the presence of iron-positive cells, visualized under light microscope and fluorescence microscope to detect FITC-positive cells.

In immunohistochemistry staining, sections were washed with PBS and incubated in 0.2% Triton X-100 in PBS for 15 min. The sections were then blocked with 3% normal goat serum and 3% bovine serum albumin, 0.2% triton X-100 in PBS for one hour and incubated in primary antibody overnight at 4 °C that included anti-mCherry (1:100; GeneTex, Taipei, Taiwan) and Ki67 (1:200; Abcam, Cambridge, UK). Primary antibodies were detected in a 1-h incubation at room temperature with secondary antibodies the appropriate Alexa Fluor 405 or 594, 647 conjugated-secondary antibodies (1:250, Invitrogen) then washed with PBS and coverslipped with mounting medium.

### 4.15. Microscope Image Acquisition

The photomicrographs shown in this study were obtained using a wide-field Olympus microscope (BX43) equipped with bright field and fluorescence microscopy. Digital image documentation was performed with a DP80 camera (Olympus, Tokyo, Japan). The quantification of fluorescence intensities was determined using ImageJ program (U.S. National Institute of Health).

### 4.16. Statistical Analysis

For in vitro studies, at least three independent experiments were performed for each statistical analysis. The Student’s *t*-test was performed to determine differences in the diameter of the hypointense areas between MSC-mCherry- and MSC-FGF21-treated animals at each distance. The Student’s *t*-test was also used to determine differences in the total hypointensities area at each time point between MSC-mCherry and MSC-FGF21-treated animals. The significant level was set at *p* < 0.05. All calculations were completed using GraphPad Prism 6 for Windows (GraphPad Software, San Diego, CA, USA).

## Supplementary Materials

The following are available online at https://www.mdpi.com/1422-0067/20/11/2624/s1.
