# Supplementary material for: Enhanced Homing of Mesenchymal Stem Cells Overexpressing Fibroblast Growth Factor 21 to Injury Site in a Mouse Model of Traumatic Brain Injury
Source: Int J Mol Sci. 2019 May 28;20(11):2624. doi: 10.3390/ijms20112624 (PMC6600548; doi:10.3390/ijms20112624)
Supplement: Supplementary file 1 [file ijms-20-02624-s001.pdf]

Shahror et al-Figure S1

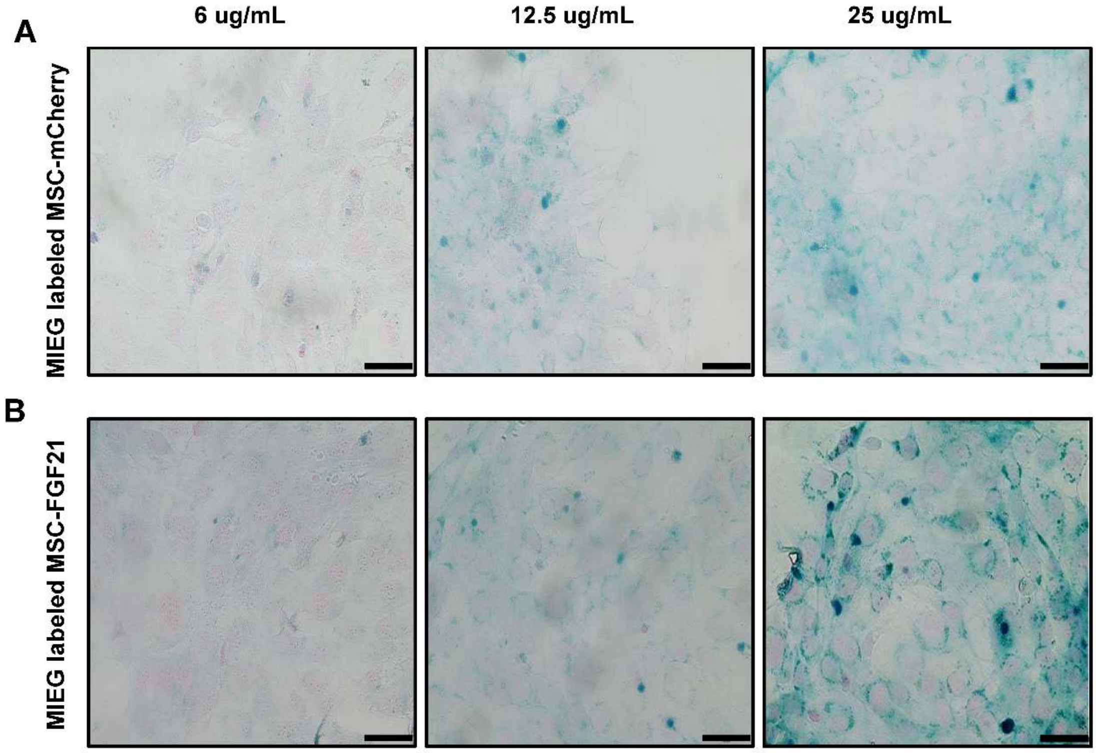

Figure S1: Prussian blue staining of MIEG labeled MSCs. Presence of MIEG in MSCs was detected by Prussian blue staining. Representative light microscope of MSC-mCherry (A) and MSC-FGF21 (B) incubated with different concentrations of MIEG for 24 h. scale bar 50 μm
